# Hyperspectral Imaging of Whole‐Cell Region for Differentiating Cervical Squamous Intraepithelial Lesion Cytology

**DOI:** 10.1002/cam4.71746

**Published:** 2026-04-03

**Authors:** Haruka Matsukawa, Keiko Yugawa, Chikai Hosokawa, Kazumi Furuichi, Kumiko Kamada, Kyoko Tanabe, Sakon Noriki, Yoshiaki Imamura, Hironobu Naiki, Kunihiro Inai

**Affiliations:** ^1^ Division of Molecular Pathology, Department of Pathological Sciences University of Fukui Fukui Japan; ^2^ Division of Surgical Pathology University of Fukui Hospital Fukui Japan; ^3^ Technology Division Panasonic Holdings Corporation Osaka Japan; ^4^ Faculty of Nursing & Social Welfare Sciences Fukui Prefectural University Fukui Japan; ^5^ Health Support Center Nagoya Institute of Technology Aichi Japan

**Keywords:** cervical cancer, cytology, differential diagnosis, hyperspectral imaging, squamous intraepithelial lesion

## Abstract

**Background:**

Cervical cytology offers a relatively safe and reliable method for cancer screening, but the tests contain vague grading criteria, such as atypical squamous cells, and cannot exclude high‐grade squamous intraepithelial lesions (ASC‐H) and atypical squamous cells of undetermined significance (ASC‐US).

**Methods:**

To explore distinct differentiation points among ASC‐H, high‐grade squamous intraepithelial lesions (HSIL), and non‐keratinizing squamous cell carcinoma (SCC), and among ASC‐US, low‐grade squamous intraepithelial lesions (LSIL), and negative for intraepithelial lesion or malignancy, hyperspectral imaging analyses were performed in cells mounted on 150 Papanicolaou‐stained specimens containing nine cervical cell types. Hyperspectral data were obtained from nuclei, cytoplasm, and whole‐cell regions in 4841 cells without overlap at 15‐nm increments in wavelength within the visible light spectrum (450–750 nm).

**Results:**

Significant differences in hyperspectral intensity were observed between ASC‐H and HSIL at 487 nm to 517 nm (*p* < 0.05), as well as among SCC at 457 nm (*p* < 0.01). Further, hyperspectral intensity differences between ASC‐US and LSIL were notable at multiple wavelengths with the lowest *p*‐value (*p* = 0.006) at 532 nm. Interestingly, quantification of hyperspectral values revealed strong correlations between nucleus‐to‐cytoplasm ratio and hyperspectral intensity value of whole cell (*R* ^2^ = 0.8339, *p* < 0.001), indicating that intensity from the whole cell provided the most informative indicator of cervical cells.

**Conclusion:**

These results suggest that hyperspectral analysis of whole cells offers a valuable tool for differentiating atypical squamous cells from other cell types.

## Graphical abstract

We present here the effectiveness of hyperspectral imaging of Papanicolaou stained cells in the differential diagnosis between ASCs and other cell types.

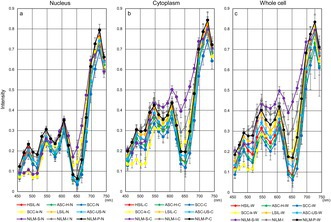

## 1 Introduction

Cervical cancer represents a formidable health challenge as the fourth most prevalent cancer among women worldwide [1]. Cervical cancer is also characterized by a significantly worse prognosis when diagnosed at an advanced stage [2]. Notably, cervical cancer ranks as the fourth leading cause of cancer‐related death among women worldwide [1]. When the lesion remains within the squamous epithelium, conical resection can achieve complete cure while preserving fertility. Pathogenetically, cervical cancer is initiated by infection of the cervical squamous epithelium with the highly carcinogenic human papillomavirus (HPV). Despite recent advances in prevention, such as HPV vaccination [3], early and highly reliable diagnosis remains central to improving patient outcomes.

In rapid screening for cervical cancer, two significant innovations were the advent of cytology for diagnosing neoplasms about 100 years ago [4] and the establishment of Papanicolaou staining in 1941, [5] leading to the current “Papanicolaou test” as a key cytodiagnostic tool [6]. In addition, liquid‐based cytology has also favored cytological diagnosis as a significant advance in recent years. Now, this standard test offers the advantage of being less invasive than biopsy and has been widely used in first‐line screening for cervical cancer. In addition, most guidelines for cervical cancer screening recommend a 3‐year interval for this cytology‐based inspection in the female population aged 25–65 years [7], since the test is relatively safe and reliable.

As an evaluation criterion, the Bethesda system has played a pivotal role in standardizing reporting, thereby facilitating the collection and analysis of global data [8]. Despite some advances, challenges persist with the inclusion of ambiguous categories for various atypical squamous cells (ASCs). In addition, inter‐rater agreement for cytology slides remains only moderate (*κ* = 0.57) [9]. Indeed, most patients with suspected high‐grade disease or showing a vague categorization such as ASCs require biopsy results to confirm a diagnosis and clarify management steps [10]. To overcome these nuisances, incorporating dual immunostaining into cytological samples has been shown to enhance the sensitivity for detecting high‐grade precancer [11]. However, this approach necessitates additional specimen preparation for immunostaining. Because the inspection is not completed on a single glass specimen slide, atypical cells such as those the examiner was unable to confirm a diagnosis for are not always present on additionally prepared specimens. Another easy approach is therefore needed to elicit further information from Papanicolaou‐stained specimens, but definitive qualitative determination from a single slide remains elusive.

In parallel with our research into molecular pathology, [12, 13] we have been engaged in collaborative medical engineering research over the past decade [14, 15, 16]. Hyperspectral imaging (HSI) is a digital technology that provides rich spatial and spectral information and extends image inspection beyond the normal limits of human perception [17]. This technology has now been applied to histopathology [18, 19, 20, 21]. The typical staining method in histopathological diagnosis uses the two colors from hematoxylin and eosin dyes. Compared to hematoxylin–eosin staining, Papanicolaou staining uses five dyes: Orange G, Eosin Y, Light Green, Bismark Brown, and Hematoxylin and produces a wider range of polychromaticity [22]. Keratinocytes are stained orange (light spectrum: 590–640 nm) by Orange G, red for the cytoplasm of keratinocytes and eosinophils (640–750 nm) by Eosin Y, blue‐green for the cytoplasm of cells with low keratinization (470–560 nm) by Light Green, and blue‐violet for nuclei (430–490 nm) by Hematoxylin. Bismarck brown stains lipids brown but does not appear in the light spectrum (Figure S1). This procedure for cytology using abundant dyes let us initiate HSI to investigate cytological specimens. Indeed, the potential of HSI in cytology remains relatively unexplored.

In this research, we first categorized cervical cell types as normal squamous cells termed negative for intraepithelial lesion or malignancy (NILM), ASCs, squamous intraepithelial lesions (SILs), and squamous cell carcinomas (SCCs). Each cell type was then subcategorized. NILMs were subcategorized as superficial NILM (NILM‐S), intermediate NILM (NILM‐I), or parabasal NILM (NILM‐P). ASCs were subcategorized as ASC of undetermined significance (ASC‐US) or ASC, cannot exclude HSIL (ASC‐H). SILs were subcategorized as low‐grade SIL (LSIL) or high‐grade SIL (HSIL). SCCs were subcategorized as keratinizing SCC (SCC‐k) or non‐keratinizing SCC (SCC). Subsequently, HSI was performed for the nucleus, cytoplasm, and whole cell for each cell type using a hyperspectral camera, followed by an exploration of spectral wavelengths that might allow differentiation among ASC‐H, HSIL, and SCC, and among ASC‐US, LSIL, and NILM‐P.

We report here the usefulness of HSI of the whole cell in differentiating ASCs from other cell types. To the best of our knowledge, this study is the first to demonstrate the effectiveness of whole‐cell HSI for differentiating among various cervical cell types.

## 2 Materials and Methods

### 2.1 Sample Collection, Preparation, and Staining

Cervical cell samples were collected from patients who visited the Department of Obstetrics and Gynecology, University of Fukui Hospital, between February 2018 and December 2023, and quickly soaked in fixative solution in PreservCyt Solution vials (Hologic, Tokyo, Japan). After overnight or longer fixation, cells were adhered to glass slides using a ThinPrep 5000 processor (Hologic) according to the manufacturer's instructions in the Department of Pathology/Histopathology, University of Fukui Hospital. Slide sections were treated with 95% ethanol for at least 60 min, then underwent Papanicolaou staining using an automated staining machine (Tissue‐Tek Prisma *Plus*; Sakura Finetech, Tokyo, Japan) as follows: 5× diluted Tissue Tech Mayer's hematoxylin (Sakura Finetech) for 16 min, 1× OG‐6 (Mutoh Chemical, Tokyo, Japan) for 6 min and 1 × EA‐50 (Mutoh Chemical) for 18 min. Following the cytological examination, the cases of ASC‐H and HSIL undergone Ki‐67 and p16 immunohisto chemical staining were extracted from electronic medical records [23]. Of the 25 ASC‐H cases undergone immunohistochemical staining, 6 were diagnosed as CIN1 and 19 as CIN2/3, respectively. Regarding the 21 HSIL cases, 20 were CIN2/3 and one was SCC using the same methods.

All research protocols were approved by the review board at the University of Fukui Hospital (No. #20210005) and conformed to the provisions of the Declaration of Helsinki. In accordance with clinical research guidelines, the Ethics Committee approved that written consent from the patient was not required because this study used only cytopathology images with no risk of identifying personal information.

### 2.2 Diagnosis of Slide Sections

Stained specimens were screened by two or more independent cytologists according to Bethesda 2014‐compliant reporting forms, followed by final diagnosis by at least one board‐certified specialist in cytodiagnosis. Twenty‐five each of NILMs, ASC‐USs, LSILs, HSILs, ASC‐Hs, and SCCs/SCC‐ks (17 SCCs and 8 SCC‐ks) were randomly selected from preserved specimens. NILMs were classified as NILM‐S, NILM‐I, and NILM‐P.

### 2.3 Hyperspectral Camera System, Image Caption, and Cell Annotation

**FIGURE 1:**
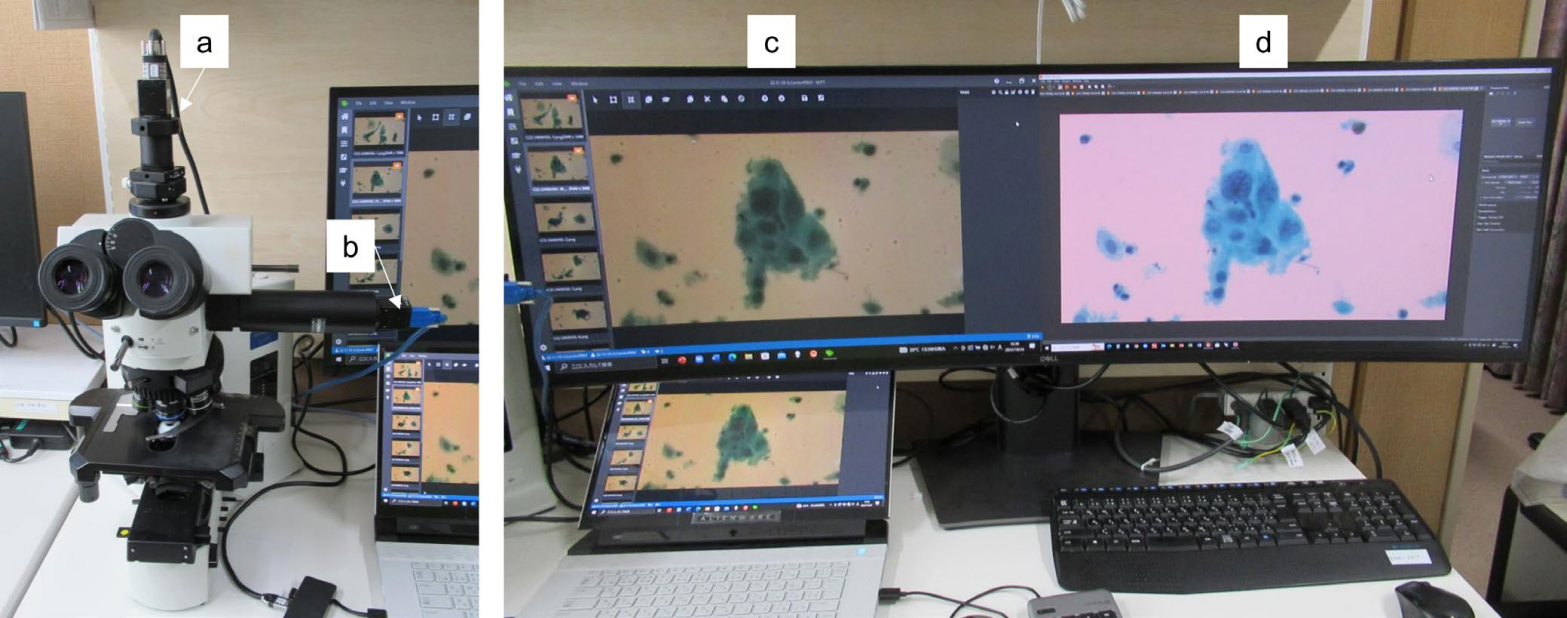
Hyperspectral camera system using this study. (a) Hyperspectral camera mounted on a BX51 optical microscope. (b) Additional digital camera coupled to the side of the microscope body for RGB images. (c, d) Representative color composite hyperspectral image (c) and its RGB image (d). Images were captured using the ×40 objective lens.

A new hyperspectral system with the world's highest sensitivity was obtained from Panasonic (Osaka, Japan) as described previously [17]. The hyperspectral camera was mounted on the top of a BX51 optical microscope (Olympus Life Science, Tokyo, Japan) using a 12‐V 100‐W halogen lamp as a light source (Figure 1a). Before developing hyperspectral images, the heat‐blocking filter was removed from the light source box to prevent attenuation of wavelengths in the 600–750 nm spectral region.

**FIGURE 2:**
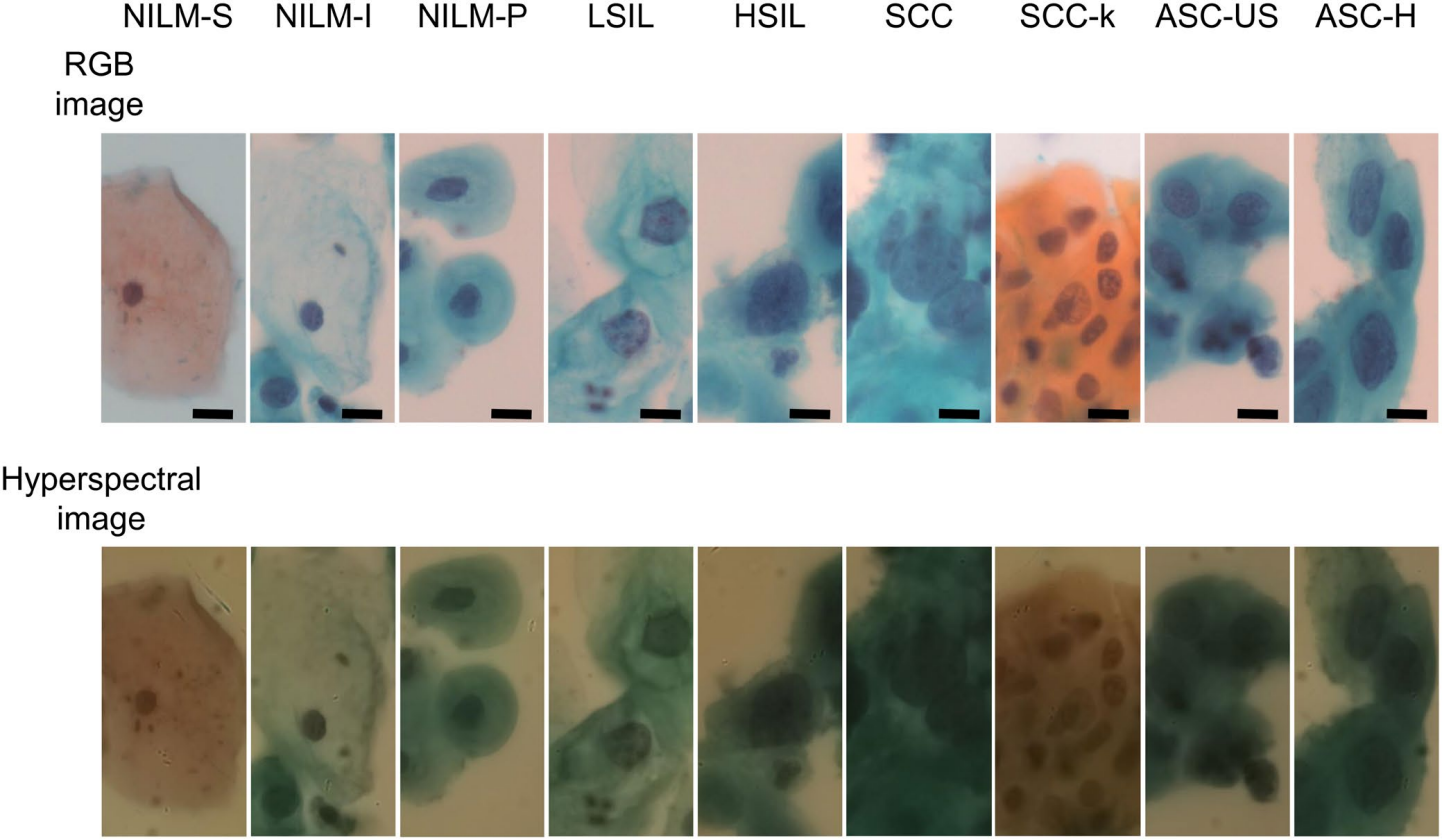
Representative RGB and hyperspectral images of 9 kinds of cell types. Representative RGB and hyperspectral images of each cell type. Upper column shows the RGB image of each cell type with Papanicolaou staining. Bottom row represents the respective hyperspectral images. Scale bar corresponds to 25 μm. ASC‐H indicates atypical squamous cells, cannot exclude high‐grade squamous intraepithelial lesions; ASC‐US, atypical squamous cells of undetermined significance; HSIL, high‐grade squamous intraepithelial lesions; LSIL, low‐grade squamous intraepithelial lesions; NILM‐I, negative for intraepithelial lesion or malignancy—intermediate squamous cell; NILM‐P, negative for intraepithelial lesion or malignancy—parabasal squamous cell; NILM‐S, negative for intraepithelial lesion or malignancy—superficial squamous cell; SCC, squamous cell carcinoma; SCC‐k, keratinizing squamous cell carcinoma.

To take the same position of Papanicolaou‐stained RGB images as that of hyperspectral images, another digital charge‐coupled device (CCD) camera was coupled to the side of the microscope body using a U‐DP1XC attachment (Olympus, Figure 1b). Hyperspectral images and RGB images were taken using the same 40× objective lens (UPlanSApo, Olympus), and the orientations of RGB images were adjusted using XIMEA CamTool software (XIMEA, Lakewood, CO) (Figure 1c,d). The hyperspectral system operates under the following conditions: a spectral range of 450–750 nm, a resolution of 2084 × 1088 pixels, an exposure time of 0.0070s, a frame rate of 85.147 frames per second (fps), and a mean dark noise reduction of 10. Hyperspectral camera image was output as a color composite image using AcquireSpectrum image processing software (Panasonic). Representative images from both Papanicolaou staining and hyperspectral cervical cell imaging are shown in Figure 2.

**FIGURE 3:**
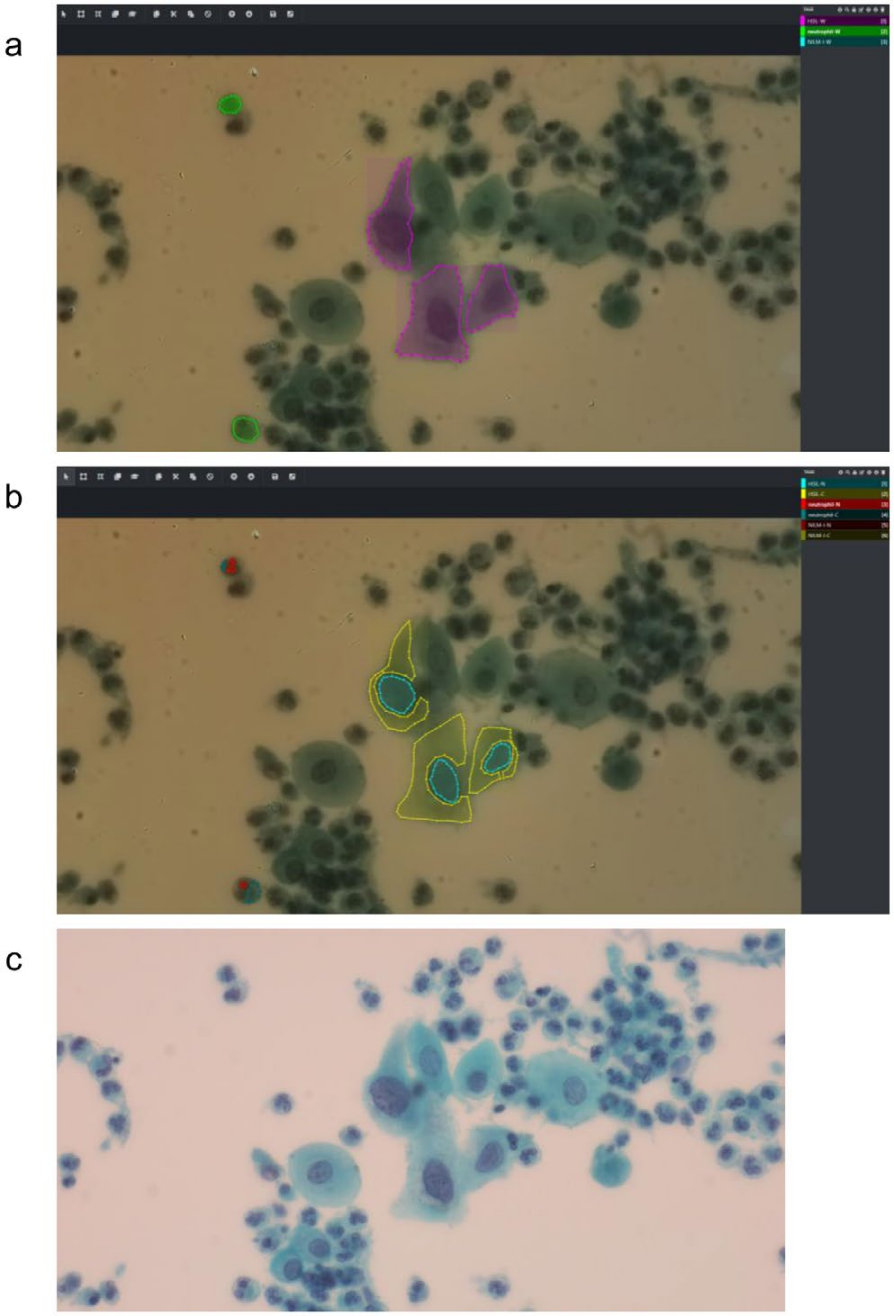
Typical annotation views from captured hyperspectral images. The VoTT annotation tool was used for annotating regions of interest (ROIs) in hyperspectral images from (a) whole cells (upper), (b) nucleus, and cytoplasm (middle). (c) Bottom row shows the Papanicolaou‐stained images.

The VoTT annotation tool (Microsoft, Redmond, WA) was used to extract regions of interest from acquired hyperspectral images, and nuclear, cytoplasmic, and whole cell hyperspectral data were obtained from the same cells (Figure 3). Cells occupying stratified regions, as the so‐called “Z stack”, were eliminated from this research. The number of cells and the interval from sample preparation to HSI selected for this study are shown in Table 1. The diagnosis of each selected cell was double‐checked by cytotechnologists and then confirmed by board‐certified cytopathologists. Data were output as the average of hyperspectral images in the region of interest (ROI). Nuclear, cytoplasmic, and whole‐cell hyperspectral data were obtained from 9 different cell components. The nucleus‐to‐cytoplasm (N/C) ratio for the annotated area was calculated for each case. The N/C ratio was obtained by dividing the area of the nucleus by the area of the whole cell.

**TABLE 1:** The precise number of cells and interval from sample preparation to hyperspectral imaging used in this study.

		NILM			ASC‐US	LSIL	ASC‐H	HSIL	Squamous cell carcinoma	
		Superficial (NILM‐S)	Intermediate (NILM‐I)	Parabasal (NILM‐P)					Non keratinizing (SCC)	Keratinizing (SCC‐k)
Total periods	Preparates	25			25	25	25	25	25	
	Cells	43	332	432	568	726	861	909	875	95
Interval until hyperspectral imaging	less than 1 year	43	332	27	240	326	287	403	190	5
	1 to 3 years			405	282	169	394	506	503	38
	more than 3 years				46	231	180		182	52

Abbreviations: ASC‐US, atypical squamous cells of undetermined significance; ASC‐H, atypical squamous cells cannot exclude HSIL HSIL, high grade squamous intraepithelial lesion; LSIL, low grade squamous intraepithelial lesion; NILM, negative for intraepithelial lesion or malignancy; SCC, squamous cell carcinoma.

### 2.4 Statistics

BellCurve for Excel version 4.01 software (Social Survey Research Information, Tokyo, Japan) was used for all statistical analyses. The Mann–Whitney U test and Spearman's rank correlation coefficient were used in this study. We expressed correlation coefficients as the coefficient of determination in this study. Values of *p* < 0.05 were accepted as statistically significant for the Mann–Whitney U test.

## 3 Results

### 3.1 Eligibility for the Samples Within the Interval Between Sample Preparation and HSI

**FIGURE 4:**
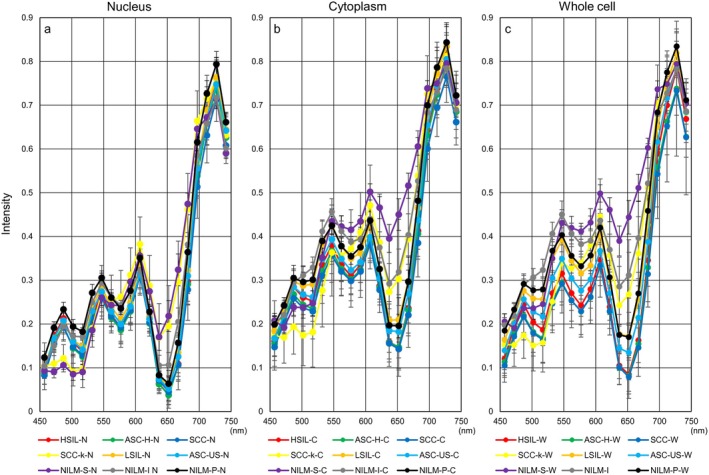
Hyperspectral intensity patterns in three cell components from all prepared samples. Hyperspectral intensity patterns among nucleus (a), cytoplasm (b), and whole cell (c) for wavelengths from 457 nm to 752 nm in each cell type. Data represent the mean ± standard deviation.

**FIGURE 5:**
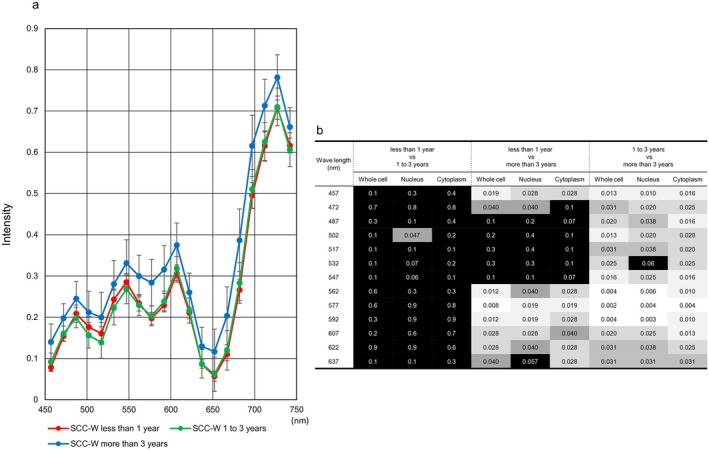
Relationship between hyperspectral intensity and the duration from sample preparation to development in the SCC. (a) The representative hyperspectral intensity of whole cells in the SCC, as measured using cytological specimens prepared more than three years prior to imaging, was elevated at all wavelengths. Red: Data from preparations taken less than one year to develop. Green: Data from sample slides taken one to three years to develop. Blue represents data from preparations taken more than three years to develop. (b) Summary of *p*‐value comparisons between two durations from sample preparation to hyperspectral image development.

Since our hyperspectral camera is highly sensitive, we first examined the suitability of the samples used in this study. Figure 4 shows the hyperspectral intensity values from three cellular components. Unexpectedly, no statistical significance was observed in intensity between ASC‐H, HSIL, and SCC (data not shown). To explore the reason, we divided the time elapsed between sample preparation and HSI acquisition into three groups—less than one year, one to three years, and three years or more—and verified the intensity in each group. The mean intensities of several wavelengths from three or more years after Papanicolaou staining were significantly higher than those from less than three years, whereas almost no statistical difference was determined between samples from less than one year and those from one to three years (Figure 5a,b).

**FIGURE 6:**
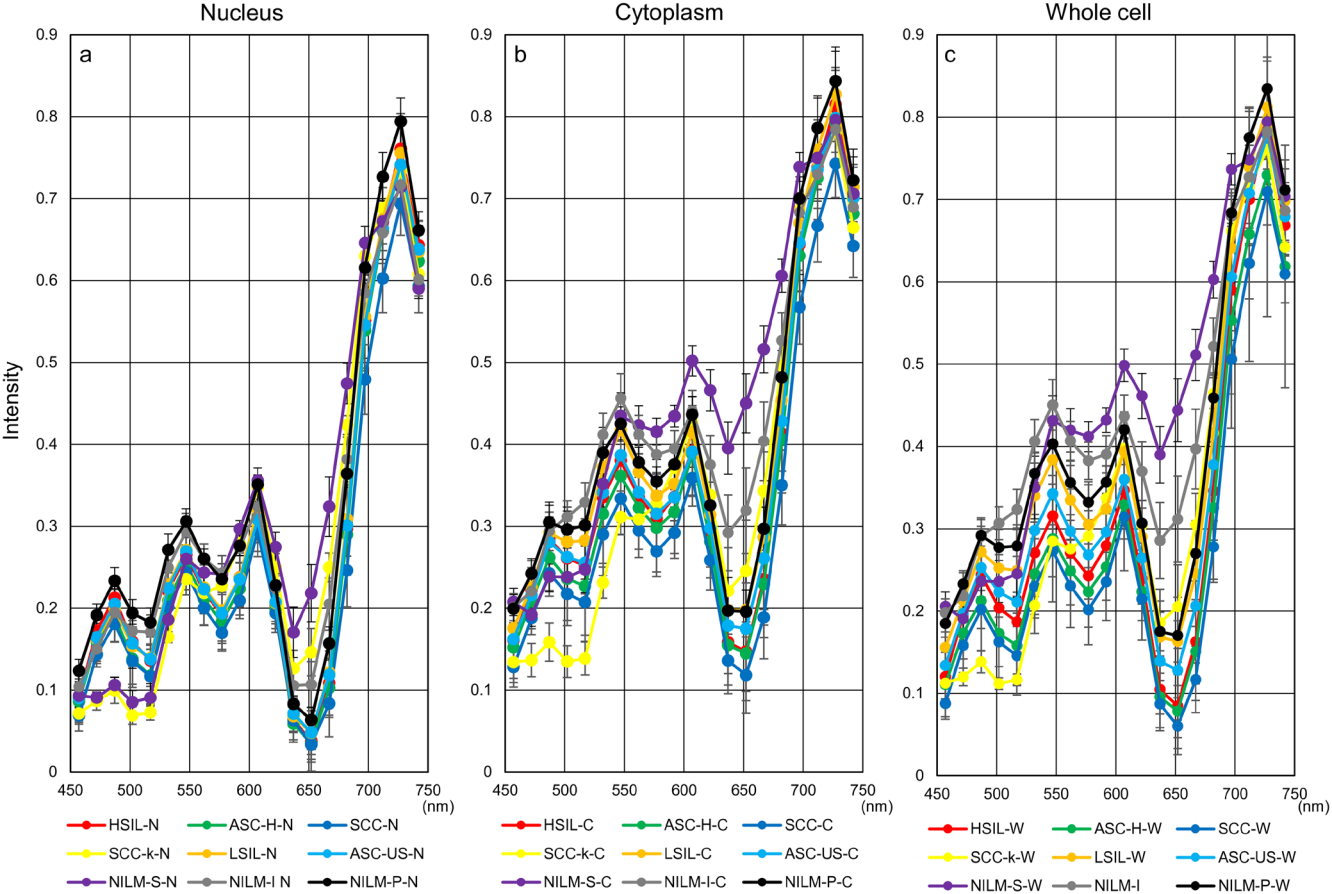
Hyperspectral intensity patterns in three cell components using samples collected less than 3 years prior to HSI. Hyperspectral intensity patterns among nucleus (a), cytoplasm (b), and whole cell (c) for wavelengths from 457 nm to 752 nm in each cell type. Data represent the mean ± standard deviation. nm indicates nanometers.

**FIGURE 7:**
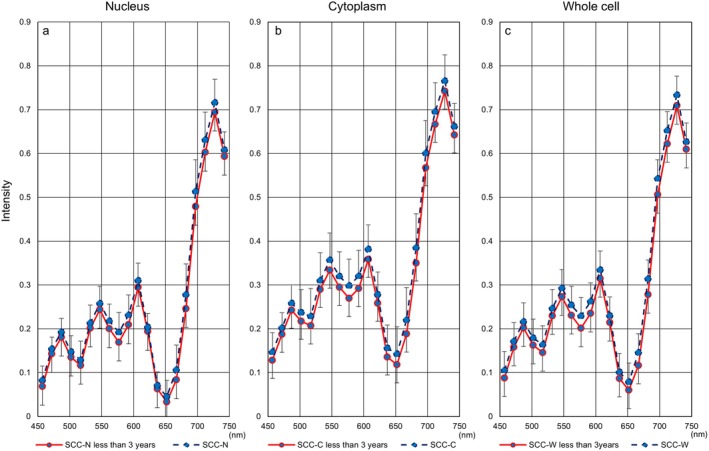
Hyperspectral intensity patterns for SCC between the entire period and the period of less than three years. The intensity pattern of the entire period (blue dotted line) showed a higher tendency than that of the less than three years (red solid line).

**FIGURE 8:**
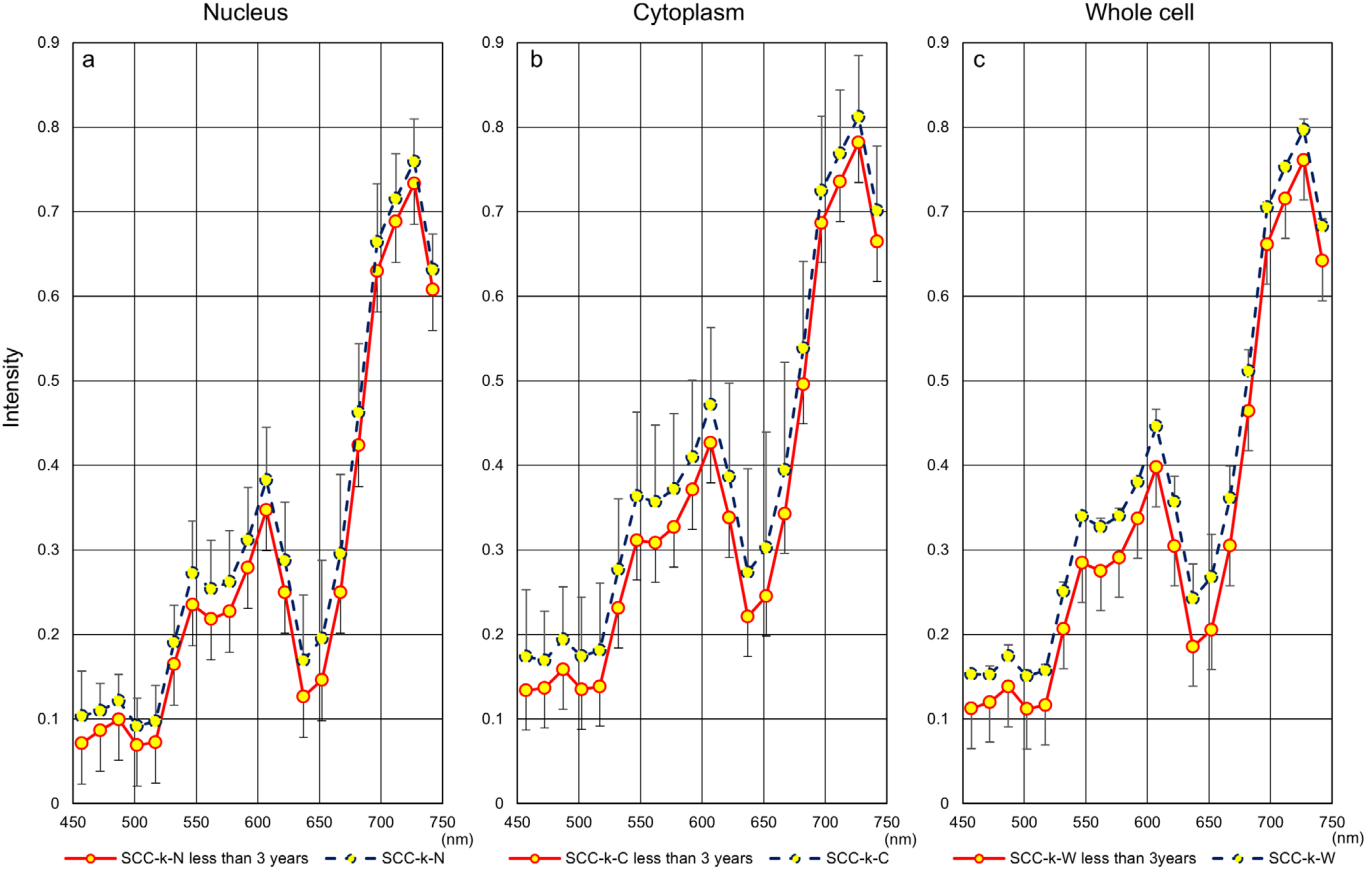
Hyperspectral intensity patterns for SCC‐k between the entire period and the period of less than three years. The intensity pattern of the entire period (blue dotted line) showed a higher tendency than that of the less than three years (red solid line).

**FIGURE 9:**
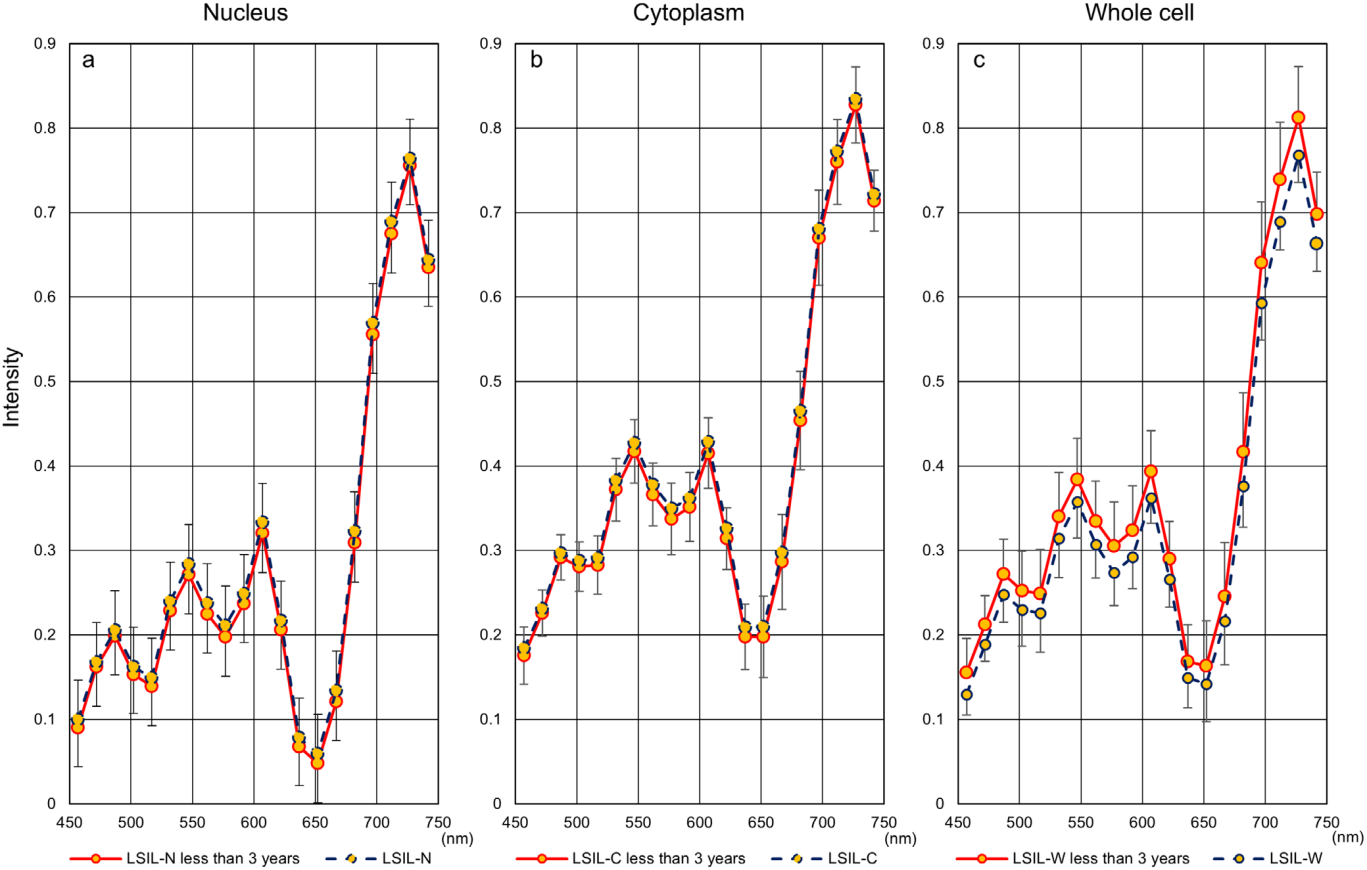
Hyperspectral intensity patterns for LSIL between the entire period and the period of less than three years. The intensity patterns of the nucleus and the cytoplasm were almost identical for the entire period (blue dotted line) and for less than three years (red solid line), respectively. However, the whole‐cell pattern for the entire period showed a lower tendency than that for less than three years.

**FIGURE 10:**
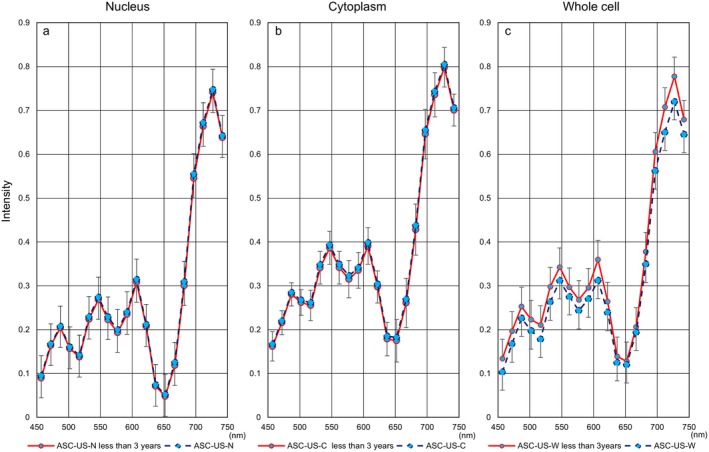
Hyperspectral intensity patterns for ASC‐US between the entire period and the period of less than three years. The intensity patterns of the nucleus and the cytoplasm were almost identical for the entire period (blue dotted line) and for less than three years (red solid line), respectively. However, the whole‐cell pattern for the entire period showed a lower tendency than that for less than three years.

**FIGURE 11:**
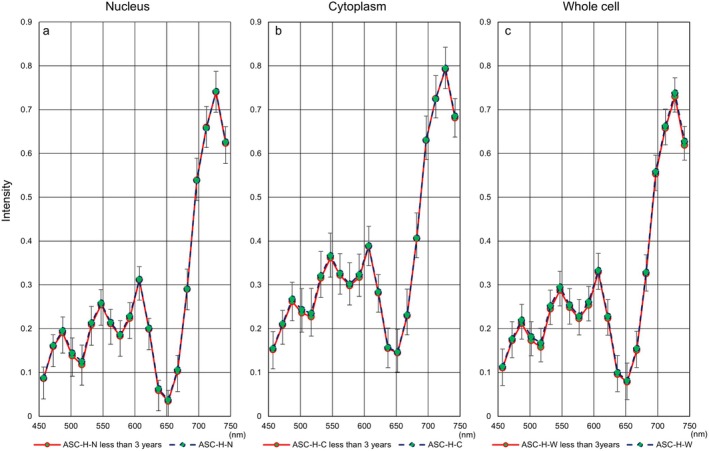
Hyperspectral intensity patterns for ASC‐H between the entire period and the period of less than three years. The intensity patterns for the entire period (blue dotted line) and for less than three years (red solid line) were almost completely identical.

Next, Figure 6 shows hyperspectral intensity patterns in three cell components using samples collected less than three years prior to HSI. We also evaluated differences in hyperspectral intensity patterns for each cell type between the entire period and the period of less than three years. HSI in whole cells showed a downward trend in more aggressive cell types (SCC: Figure 7, SCC‐k: Figure 8) and an upward trend in relatively lower‐growth types (LSIL: Figure 9, ASC‐US: Figure 10) when intensity data was collected from cell samples over three years ago. ASC‐H exhibited similar HSI patterns over the entire period and the period of less than three years (Figure 11), indicating that no consistent trend was observed. Therefore, samples for which more than three years had elapsed between the Papanicolaou stain and HSI were eliminated from this study.

### 3.2 Higher Resolution Potential of Intensity Patterns of Hyperspectral Images From Whole Cells

The intensity patterns of HSI for nucleus, cytoplasm, or whole cell, capable of clarifying the characteristics in 9 different cervical cell types as much as possible, are shown in Figure 6. Briefly, intensity in each cell type showed 4 convex peaks at wavelengths of 487, 547, 607, and 727 nm and 3 concave peaks at wavelengths of around 517, 577, and 637 nm, respectively (Figure 6a–c). The spectral pattern of nuclei, regardless of cell type, was relatively concentrated in narrow ranges compared to that of cytoplasm and whole cells (Figure 6a). In addition, intensities from nuclei were distributed at lower levels than those from the cytoplasm and whole cell. Intensities from cytoplasm were generally higher than those from nuclei and were particularly prominent in NILM‐S and NILM‐I (Figure 6b). Interestingly, hyperspectral patterns appeared broadly divisible into three major groups using analyses from whole cells (Figure 6c). The first group with the highest intensities comprised the three normal cervical cell types including NILM‐S, NILM‐I, and NILM‐P. Intensities within these three NILMs may be ordered by the proliferative faculty of the cells. On the other hand, the three cell types showing the lowest intensities were HSILs, SCCs, and ASC‐Hs, showing malignant potential. The remaining LSIL and ASC‐US were located between the above two groups. Regarding SCC‐k, the intensity pattern was at the lowest level at wavelengths less than 502 nm, but increased sharply around 547–607 nm and reached an overall intermediate level in the high‐wavelength range, as for NILM‐P.

Table S1 shows the mean and standard deviation of each hyperspectral intensity obtained from nuclear, cytoplasmic, and whole cell at each wavelength. These are the specific measured hyperspectral intensities obtained from 9 different cell components in this study. The values in parentheses represent the 95% confidence interval.

### 3.3 Three Classifications of Cervical Cells Based on N/C Ratio

**FIGURE 12:**
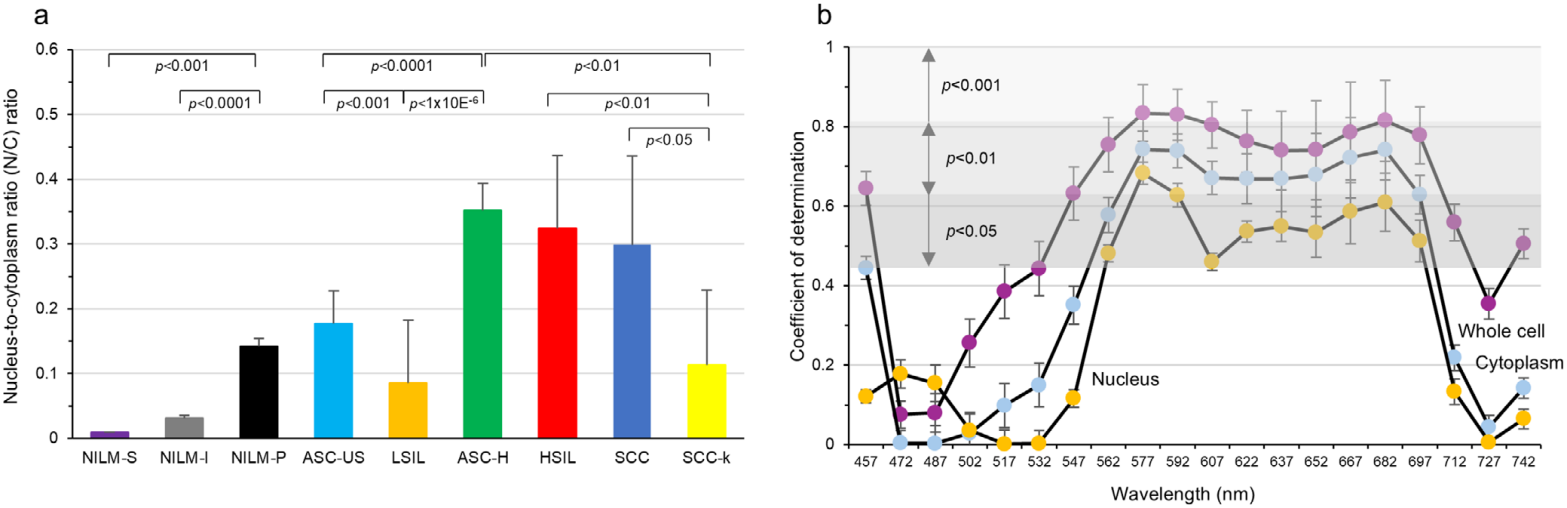
N/C ratio in each cell type and correlation between N/C ratio and hyperspectral intensity. (a) N/C ratio in each cervical cell type. The N/C ratio was obtained by dividing the area of the nucleus by the area of the whole cell. Data represent the mean ± standard deviation. (b) Correlation between primary correlation and N/C ratio for nucleus, cytoplasm, and whole cell at each wavelength. Y axis shows the coefficient of determination determined at each wavelength. nm indicates nanometers.

As shown in Figure 12a, NILM‐S without mitotic potency presenting in the upper part of the squamous epithelium showed a markedly low N/C ratio. NILM‐I representing cells below the NILM‐S also showed a low ratio of about 0.05. In comparison, the value for NILM‐P, a mitotically competent of normal epithelium, was increased to levels similar to those of LSIL and ASC‐US. These three cell types seemed to form a second group with a mean N/C ratio of about 0.13. ASC‐H, HSIL, and SCC could be summarized as a third group with an N/C ratio > 0.3, including highly suspicious lesions of malignancy above HSIL (ASC‐H, HSIL, SCC). SCC‐k showed an N/C ratio between ASC‐US and LSIL but may not be included in any group because of the quite different cytoplasm color from other cell types. The N/C ratio by annotation may thus be divided into three groups, excluding SCC‐k.

**FIGURE 13:**
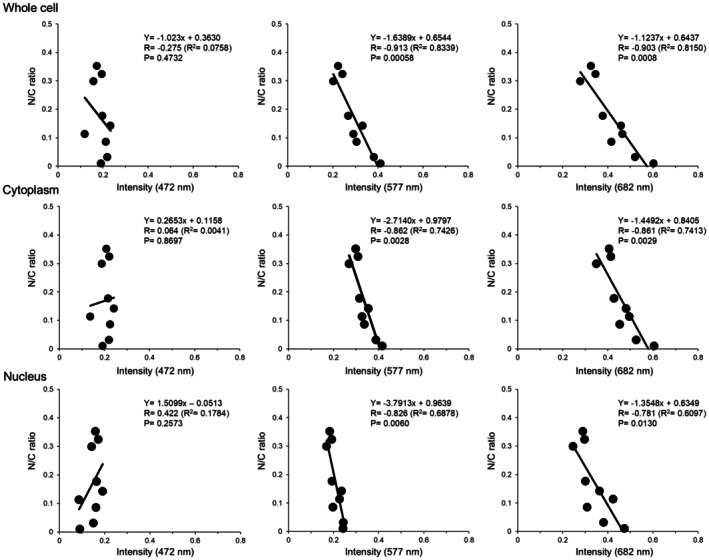
The relationship between N/C ratio and intensity of representative wavelengths. Correlation coefficient between N/C ratio and intensity of the specific wavelength, analyzed using Spearman's rank method. Representative data of 472 nm, 577 nm, and 682 nm from whole cell, cytoplasm, and nucleus are shown in this slide. The highest correlation coefficient values were obtained from the intensity results of hyperspectral imaging in whole cells.

Next, we examined which of the three parameters best correlated with N/C ratio. Figure 12b depicts the association between coefficient of determination and N/C ratio in each wavelength of the three types of analysis. Intensity for whole cells decreased from low N/C ratio to high N/C ratio within the wavelength range of 577–607 nm. Whole cell intensity comprises the intensity of the cytoplasm and nucleus, and the higher the N/C ratio, the stronger the effect of a lower‐intensity nucleus. However, the intensity of the cytoplasm also decreased in the order of the N/C ratio at 607 nm. The coefficient of determination derived from whole cell data was higher than those from cytoplasm and nucleus, and correlation coefficients for whole cell were above −0.9 at 577 nm, 592 nm, and 682 nm (Figure 12b and Figure 13). Based on these results, the following study was conducted using whole cell hyperspectral data.

### 3.4 Effectiveness of Analyzing Around a 500‐nm Wavelength Hyperspectral Images for Differentiating Between ASC‐H and HSIL

**FIGURE 14:**
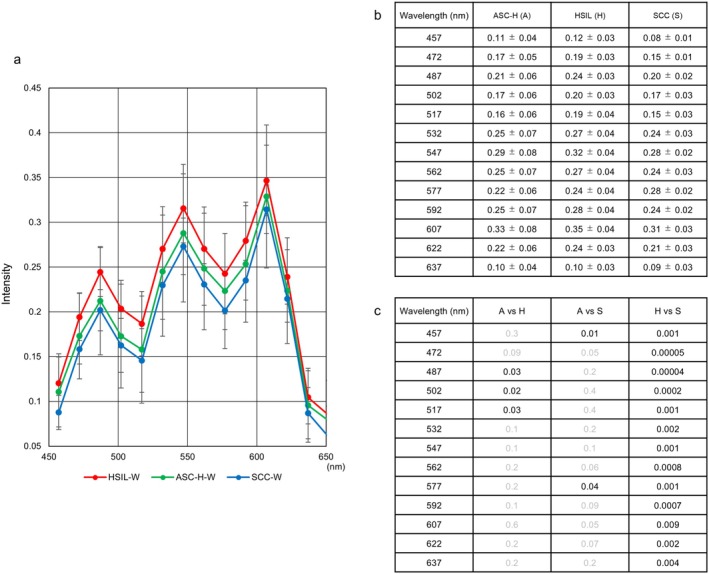
Differential diagnostic points between ASC‐H and HSIL or SCC by hyperspectral intensity. (a) Magnified view of hyperspectral patterns of whole cell from 457 nm to 637 nm among ASC‐H, HSIL, and SCC. (b) Intensity data between ASC‐H and HSIL or SCC. Data represent the mean ± standard deviation. (c) *p*‐values between ASC‐H (A) and HSIL (H) or SCC (S). Statistical analyses were performed using the Mann–Whitney U test. Gray numbers indicate no statistical significance.

To investigate whether hyperspectral image analyses could better differentiate between ASC‐H and HSIL, we compared hyperspectral patterns between these cell types. Figure 14a shows a magnified view of the hyperspectral pattern from 457 nm to 637 nm of whole cells among ASC‐H (green line), HSIL (red line), and SCC (blue line). Compared to the intensity of ASC‐H, that of HSIL showed a significantly higher trend from 487 nm to 517 nm. Meanwhile, the levels of SCC revealed a significant decline at 457 nm (Figure 14b,c). Regarding the intensity levels between HSIL and SCC, HSIL consistently demonstrated a significant increase from 457 nm to 577 nm (Figure 14b,c).

### 3.5 Support for Differentiation Among ASC‐US, LSIL, and NILM‐P Using HSI

**FIGURE 15:**
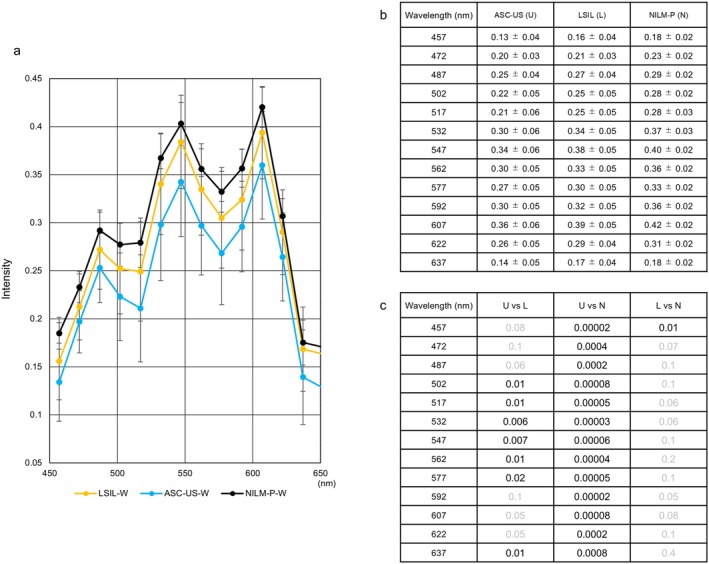
Differentiating between ASC‐US and LSIL or NILM‐P by hyperspectral intensity. (a) Magnified view of hyperspectral patterns from whole cells at wavelengths from 457 nm to 637 nm among ASC‐US, LSIL, and NILM‐P. (b) Intensity data between ASC‐US and LSIL or NILM‐P. Data represent the mean ± standard deviation. (c) *p*‐values between ASC‐US (U) and LSIL (L) or NILM‐P (N). Statistical analyses were performed using the Mann–Whitney U test. Gray numbers indicate no statistical significance.

Similar to ASC‐H, a morphologically based category of ASC‐US has been created to distinguish cases of round or ovoid NILM or suspected LSIL where the presence of koilocytosis cannot be fully confirmed. We examined the spectrum of atypical cells in specimens diagnosed as ASC‐US for differences from NILM‐P, and also for differences from LSIL. A graph of these cell portions enlarged from Figure 6 (whole cells) is shown in Figure 15a. Each spectral pattern of ASC‐US between wavelengths of 502 nm and 577 nm was significantly lower than those of LSIL (Figure 15b,c). Additionally, hyperspectral intensity from 457 nm to 607 nm proved useful for differentiating between NILM and ASC‐US (Figure 15b,c).

### 3.6 Useful Wavelengths for Differentiating ASCs From Other Cell Types by HSI

Then, we explored the best wavelength for differentiating ASCs from other cell types by HSI. The results are summarized in Table 2. In this study, *p*‐values were not adjusted using approaches such as the Holm method for multiple testing because each raw value represented an independent condition and the cumulative risk ratio did not exceed 1.0 [24, 25]. Overall, identification capabilities using whole cell hyperspectral data tended to be superior to those using nucleus or cytoplasm. The wavelengths of 502 nm (range 487 nm to 517 nm) and 457 nm were optimal for differentiating ASC‐H from HSIL or SCC, respectively. As for ASC‐US, 532 nm (range 502 nm to 577 nm) was advantageous for differentiating it from LSIL. For identifying ASC‐US from NILM‐P, any wavelength between 457 nm and 637 nm was available, of which between 502 nm and 607 nm were almost equally effective.

**TABLE 2:** Summary of *p*‐value comparisons between two different cervical cell types in each condition.

Wave length (nm)	ASC‐H vs. HSIL			ASC‐H vs. SCC			HSIL vs. SCC			ASC‐US vs. LSIL			ASC‐US vs. NILM‐P			LSIL vs. NILM‐P		
	Whole cell	Nucleus	Cytoplasm	Whole cell	Nucleus	Cytoplasm	Whole cell	Nucleus	Cytoplasm	Whole cell	Nucleus	Cytoplasm	Whole cell	Nucleus	Cytoplasm	Whole cell	Nucleus	Cytoplasm
457	0.3	0.6	0.4	0.018	0.038	0.023	0.001	0.017	0.002	0.08	0.9	0.2	0.00002	0.00005	0.0002	0.012	0.00002	0.029
472	0.09	0.07	0.07	0.052	0.07	0.021	0.00005	0.0001	0.0002	0.1	0.9	0.3	0.0004	0.001	0.002	0.07	0.00006	0.1
487	0.037	0.016	0.026	0.2	0.4	0.1	0.00004	0.00007	0.0001	0.06	0.8	0.2	0.0002	0.001	0.012	0.1	0.00002	0.3
502	0.022	0.026	0.035	0.4	1	0.2	0.0002	0.003	0.001	0.019	0.7	0.07	0.00008	0.0001	0.001	0.1	0.00002	0.2
517	0.037	0.028	0.049	0.4	0.9	0.3	0.001	0.021	0.004	0.012	0.4	0.06	0.00005	0.00004	0.001	0.06	0.00001	0.1
532	0.1	0.09	0.1	0.2	0.6	0.1	0.002	0.023	0.005	0.006	0.3	0.033	0.00003	0.00006	0.0009	0.06	0.000006	0.2
547	0.1	0.1	0.1	0.1	0.3	0.1	0.001	0.008	0.0002	0.007	0.4	0.017	0.00006	0.00007	0.001	0.1	0.00004	0.6
562	0.2	0.2	0.2	0.06	0.2	0.06	0.0008	0.011	0.002	0.012	0.5	0.036	0.00004	0.00003	0.0005	0.2	0.00004	0.5
577	0.2	0.3	0.4	0.041	0.1	0.032	0.001	0.032	0.003	0.027	0.5	0.09	0.00005	0.00003	0.0006	0.1	0.00003	0.1
592	0.1	0.1	0.1	0.09	0.1	0.044	0.0007	0.005	0.0007	0.1	0.7	0.3	0.00002	0.00001	0.0003	0.055	0.00006	0.1
607	0.6	0.8	0.9	0.056	0.07	0.018	0.009	0.06	0.018	0.057	0.3	0.1	0.00008	0.0009	0.0005	0.08	0.0007	0.1
622	0.2	0.3	0.8	0.07	0.3	0.06	0.002	0.030	0.012	0.057	0.8	0.1	0.0002	0.001	0.001	0.1	0.001	0.1
637	0.2	0.1	0.6	0.2	0.5	0.09	0.004	0.5	0.030	0.019	0.7	0.07	0.0008	0.005	0.010	0.4	0.001	0.8

*Note:* Each box was colored according to the *p*‐value: white with < 0.01, faint gray with 0.01 ≤ < 0.02, light gray with 0.02 ≤ < 0.03, gray with 0.03 ≤ < 0.04, dark gray with 0.04 ≤ < 0.05, and black with 0.05 ≤ .

Abbreviations: ASC‐US, atypical squamous cells of undetermined significance; ASC‐H, atypical squamous cells cannot exclude HSIL; HSIL, high grade squamous intraepithelial lesion; LSIL, low grade squamous intraepithelial lesion; NILM‐P, parabasal negative for intraepithelial lesion or malignancy; SCC, non keratinizing squamous cell carcinoma.

On the other hand, any wavelength between 457 nm and 637 nm could be used to differentiate HSIL from SCC. Of these wavelengths, those between 472 nm and 502 nm showed almost similar effect in detecting HSIL and SCC. Discrimination between LSIL and NILM‐P was equally possible with any nuclear wavelength between 457 and 607 nm.

### 3.7 Immunohistochemistry‐Based Validation Analyses of Hyperspectral Imaging

**FIGURE 16:**
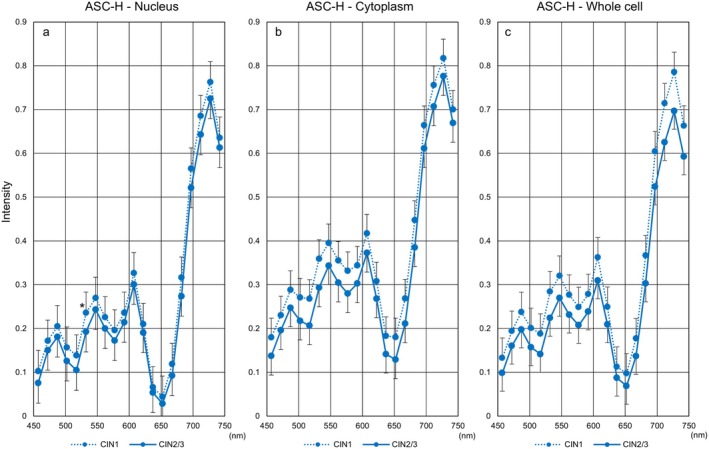
Relevance between hyperspectral analyses of ASC‐H and histological diagnosis by immunohistochemical staining. Hyperspectral intensity patterns in the nucleus (a), cytoplasm (b), and whole cell (c) in ASC‐H cases diagnosed as CIN1 (dotted line) and those diagnosed as CIN2/3 (solid line) by immunohistochemistry of tissue sections. CIN, cervical intraepithelial neoplasia. *p < 0.05.

Recent advancements in histological diagnosis using Ki‐67 and p16 immunostaining have enabled the classification of cervical intraepithelial neoplasia (CIN)1 or CIN2/3 subtypes following cytological screening of ASC‐H. Therefore, we further investigated the validity of hyperspectral imaging patterns among cases initially classified as ASC‐H by cytology and subsequently diagnosed as CIN1 or CIN2/3 by immunohistochemistry of cervical tissue. As shown in Figure 16, histological CIN1 (dotted line) cases exhibited higher hyperspectral intensity than CIN2/3 (solid line) cases in the nucleus, cytoplasm, and whole cell. The 532 nm intensity in the nucleus was statistically significant (*p* < 0.05).

**FIGURE 17:**
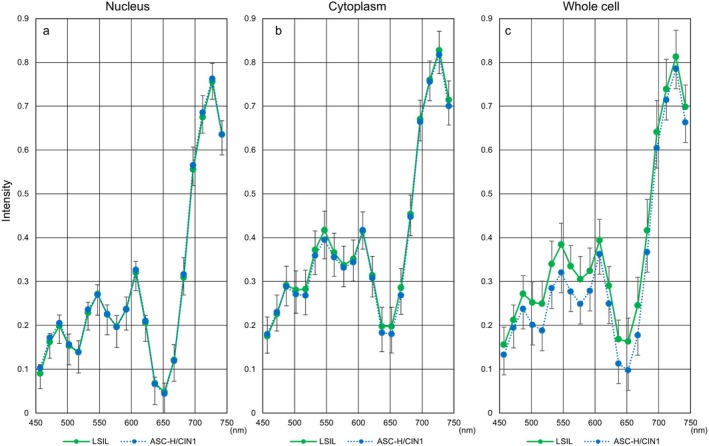
Comparison of hyperspectral intensity between LSIL and ASC‐H/CIN1 cases. Hyperspectral intensity patterns in the nucleus (a), cytoplasm (b), and whole cell (c) in LSIL (green solid line) and ASC‐H/CIN1 cases (blue dotted line).

**FIGURE 18:**
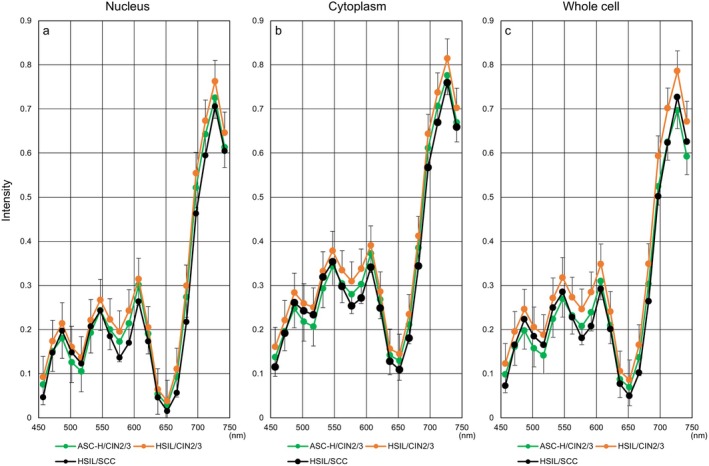
Comparison of hyperspectral intensity among ASC‐H/CIN2/3, HSIL/CIN2/3, and HSIL/SCC cases. Hyperspectral intensity patterns in the nucleus (a), cytoplasm (b), and whole cell (c) in ASC‐H/CIN2/3 (green), HSIL/CIN2/3 (orange), and HSIL/SCC (black) cases.

Next, we compared the hyperspectral intensity between LSIL and ASC‐H/CIN1 (Figure 17). Unexpectedly, the intensity of LSIL tended to be higher than that of ASC‐H/CIN1. Meanwhile, the intensities of the nucleus and the cytoplasm almost overlapped in the two conditions. Furthermore, intensity values derived from ASC‐H/CIN2/3 (green) were intermediate or proximal to those of HSIL/CIN2/3 (orange) and HSIL/SCC (black) (Figure 18).

## 4 Discussion

Basically, three components of the cell can be analyzed by hyperspectral intensity: the nucleus, cytoplasm, and whole cell. However, most hyperspectral studies have analyzed images from the nucleus [7, 8, 9] and few comparative studies have examined which of the nuclear, cytoplasmic, or whole cell hyperspectral images are most effective for pathological and cytological diagnosis. The present study validated the effectiveness of the three cellular components of 9 cervical cell types as targets of hyperspectral analysis of the same cells and found that the whole cell provided the best resource for HSI. Intensity patterns from whole cells were clearly separable into three groups, excluding SCC‐k, corresponding to the results of grouping by N/C ratio. The concept of N/C ratio is a classical morphological evaluation method using the areas of the cell nucleus and cytoplasm and is used as a simple way to estimate between benign and malignant cells. In general, the higher the N/C ratio, the more immature the cells are; this has been used as a convenient method of identifying leukemic blasts. Outside of hematology, the N/C ratio is also higher in cells representing immature phenotypes, including cervical cells. In addition, the first‐order correlation between N/C ratio and intensity levels from whole cells was a better fit than those from nucleus or cytoplasm, and the correlation coefficient reached −0.913 (R^2^ = 0.8339). However, except for NILM‐S and NILM‐I, there were no significant differences between NILM‐P, LSIL, and ASC‐US, or between HSIL, SCC, and ASC‐H, making them difficult to use for differential diagnosis. These results suggest that among nuclear, cytoplasmic, and whole cell analyses, that using hyperspectral intensity extracted from whole cells best reflected the degree of cellular biological malignancy.

Despite the exploration of specific biomarkers to improve the diagnostic accuracy of cervical cytology, particularly for ASC‐H, HSIL, and SCC, interobserver agreement has remained moderate, indicating a need for improved reproducibility [9]. After confirming that whole cell hyperspectral analysis best corresponded to the N/C ratio, we investigated the specific wavelengths capable of differentiating between cell groups and found that one or more intensity values within the range of 487 nm to 517 nm and 457 nm could be available for hyperspectral analysis to differentiate between ASC‐H and HSIL, and between ASC‐H and SCC, respectively.

The first‐line treatment for HSIL and SCC is essentially conization as long as the lesion remains within the squamous epithelium. However, this HSI study demonstrated a statistically significant distinction between HSIL and SCC. Our results also support the knowledge that HSIL and SCC have clear differences in definition. In contrast to HSIL and SCC, ASC‐H is an ambiguous cytological category for which the cytological changes are suggestive of HSIL but insufficient for definitive interpretation. One reason for this is that the diagnosis depends solely on morphological analysis. To overcome this disadvantage, high‐risk HPV testing and multiple immunostainings, including for p16 and Ki‐67 antibodies, are preferably used to increase the sensitivity of cytological diagnosis, [26, 27] but additional testing is costly and time‐consuming. In addition, preparation of new specimen slides is needed for cytological analysis from the remaining specimen. However, these do not always contain the atypical cells seen on the original Papanicolaou‐stained slide. Fortunately, our hyperspectral camera system is applicable to the original Papanicolaou‐stained slides, so spectral patterns can be extracted directly from the same atypical cells initially identified on microscopy, without any pre‐processing. In addition, the hyperspectral camera system is capable of resolving the visible light spectrum into 20 different wavelengths for analysis, meaning that 20 different feature values can be extracted from a single hyperspectral measurement.

Interestingly, when ASC‐H cases were classified as CIN1 or CIN2/3 through immunohistochemical staining for pathological diagnoses, the hyperspectral intensities in the nucleus, cytoplasm, and whole cell could be clearly distinguished according to biological malignancy. Additionally, the nuclear and cytoplasmic levels of CIN1 corresponded well to those of LSIL. Furthermore, the hyperspectral intensities of CIN2/3 cases in ASC‐H were similar to those of CIN2/3 and SCC cases derived from HSIL. These results suggest that hyperspectral analysis in ASC‐H cases may provide findings comparable to those obtained by immunohistochemical staining in cervical histology. However, the intensity of LSIL in the whole cell was higher than that of CIN1 derived from ASC‐H, indicating a dissociation between the two cell types. While this study could not identify the reasons for this deterioration, it is possible that each cell type has different staining properties and a specific N/C ratio.

Similarly, the differentiation of ASC‐US, with putative but incomplete morphological changes due to HPV infection, from NILM‐P and LSIL was facilitated by hyperspectral analyses of the three cell types. This probably allowed for complex spectral analysis and clear separation of intensities among the nine types of cervical cells. Importantly, the hyperspectral intensity of samples stained more than three years ago demonstrated three distinct patterns of higher (SCC and SCC‐k), equal (ASC‐H), or lower (LSIL and ASC‐US) than that of samples stained within three years, indicating that multiple degeneration processes may exist depending on cellular proliferative potential. Deterioration such as sample discoloration has been reported in HE‐ and Papanicolaou‐stained specimens [28, 29]. Thus, while cervical cytology is a promising target for hyperspectral camera analysis, our results underscore the importance of acquiring HSI within three years of sample preparation.

The N/C ratio is a parameter obtained by dividing the nuclear area by the cytoplasmic area. It is used as a convenient marker for evaluating cell maturity, renal epithelial dysfunction, and as a predictor of dysplasia and malignancy of pancreatic, cervical, and hematological neoplasms [30, 31, 32, 33]. Despite its simplicity and versatility, N/C ratio measurement requires manual work using image analysis software such as ImageJ. Since the N/C ratio of the eight types of cervical cells other than SCC‐k showed a tendency to be classifiable into three major groups according to the N/C ratio of the whole cell, we investigated associations between N/C ratio and hyperspectral intensities. As expected, N/C ratio showed a linear correlation with hyperspectral intensity with a high coefficient of determination, particularly at wavelengths of 577, 592, and 682 nm. The mechanism of action is unclear, but the hyperspectral intensity values corresponding to the green to yellow‐green and red regions may correlate well with the ratio between nuclear and cytoplasmic permeability of the Papanicolaou stain. Because few simplified methods have been described for estimating N/C ratio, hyperspectral images at specific wavelengths may allow favorable estimation of this ratio in several cytological analyses using Papanicolaou staining.

From a perspective of differential diagnosis, wavelengths ranging from 487 nm to 517 nm or 457 nm were useful for distinguishing between ASC‐H and HSIL or SCC. Specifically, ASC‐H has lower Hematoxylin and Light Green intensity than HSIL, implying lower transmittance of these dyes. The color tone of Hematoxylin was more concentrated in SCC than in ASC‐H. These results indicate nuclear and cytoplasmic differences are important for distinguishing features. Additionally, cytoplasmic wavelengths from 577 nm to 607 nm were notable for discriminating between ASC‐H and SCC cells. Perhaps, ASC‐H cells contain mature keratinized cytoplasm components. Regarding the relationship between HSIL and SCC, it is likely that the two cell types differ more in their cytoplasm than in their nuclei. For ASC‐US, the HSI in the 502–577 nm range was important for distinguishing it from LSIL, showing Light Green dyeability is critical. NILM‐P had critical nuclear features versus ASC‐US and LSIL, with bright nuclear staining as a hallmark. By using these observation points, we expect to reduce inter‐observer variability in ASC‐H/ASC‐US diagnoses.

Recent advances in digital technology have also been applied to artificial intelligence (AI) diagnosis of cervical cytology [34, 35, 36], indicating that AI discrimination may be superior to the HSI technique. However, some reports mention that the sensitivity of AI to discriminate ASC‐US or ASC‐H remains between 0.5 and 0.7. This may be because the definition of ASCs is ambiguous, which may lead to bias in the AI training data that many researchers have spent a long time generating. On the other hand, we present here that several wavelengths could discriminate between ASC‐H and HSIL/SCC, and ASC‐US and LSIL/NILM‐P. These results do not indicate that either AI or HSI has an advantage, but rather that digital diagnostic technology is progressing by complementing each other to become a digital diagnostic technology.

This study showed several limitations. First, the study was based on a small number of cell specimens from a single institution. Second, specimens were limited to squamous lesions of the cervix, so it is unclear whether these results are applicable to other cytological specimens. Third, this was a retrospective study. Fourth, HSI of stratified cells was not evaluated in this research. Finally, this study lacks comparative analysis. However, this study gives the key points and the specific ROI within cells that could distinguish ASC‐H from SCC and HSIL, and ASC‐US from LSIL and NILM‐P, from the perspective of color tone. Thus, we expect that the convenience and utility of HSI for cytologic diagnosis will be clarified when whole‐cell HSI studies are conducted at multiple institutions.

In conclusion, we have demonstrated the feasibility of differentiating cervical squamous cell lesions using spectral analysis of whole cells with a high‐sensitivity hyperspectral camera. This study also supports other findings that hyperspectral analysis is useful for identifying various diseases and tissues [21]. Cervical cytology is known as the most effective cancer‐prevention measure in history [27]. Therefore, although further studies are needed to confirm the present results, we believe that this HSI study will open avenues for future innovations in the realm of cervical cancer diagnosis and treatment.

## Author Contributions

**Haruka Matsukawa:** data curation (equal), investigation (equal), visualization (equal), writing – original draft (equal). **Keiko Yugawa:** data curation (equal), resources (equal), software (equal), visualization (equal). **Chikai Hosokawa:** resources (equal), software (equal). **Kazumi Furuichi:** investigation (equal). **Kumiko Kamada:** investigation (equal). **Kyoko Tanabe:** investigation (equal). **Sakon Noriki:** supervision (equal), writing – review and editing (equal). **Yoshiaki Imamura:** investigation (equal), supervision (equal). **Hironobu Naiki:** supervision (equal), writing – review and editing (equal). **Kunihiro Inai:** conceptualization (equal), formal analysis (equal), funding acquisition (equal), visualization (equal), writing – review and editing (equal).

## Funding

This work was supported by Panasonic Holdings Corporation.

## Ethics Statement

Approval of the research protocol by an Institutional Review Board: This study was reviewed and approved by the Ethics Committee of University of Fukui Hospital (approval no. #20210005) and performed in accordance with the Declaration of Helsinki. In accordance with clinical research guidelines, the Ethics Committee approved that written consent from the patient was not required because this study used only cytopathology images with no risk of identifying personal information.

## Consent

The authors have nothing to report.

## Conflicts of Interest

Kunihiro Inai received a research grant from Panasonic Holdings Corporation. Other authors declare no conflicts of interest.

## Supporting information


**Supplementary Figure S1:** Supporting information.

**Supplementary Table 1** Hyperspectral intensity data of nine different cell components in each wavelength.

## Data Availability

The data that supports the findings of this study are available in the Supporting Information of this article.
