# Supplementary figures and images for: Hyperspectral Imaging of Whole‐Cell Region for Differentiating Cervical Squamous Intraepithelial Lesion Cytology
Source: Cancer Med. 2026 Apr 3;15(4):e71746. doi: 10.1002/cam4.71746 (PMC13052316; doi:10.1002/cam4.71746)

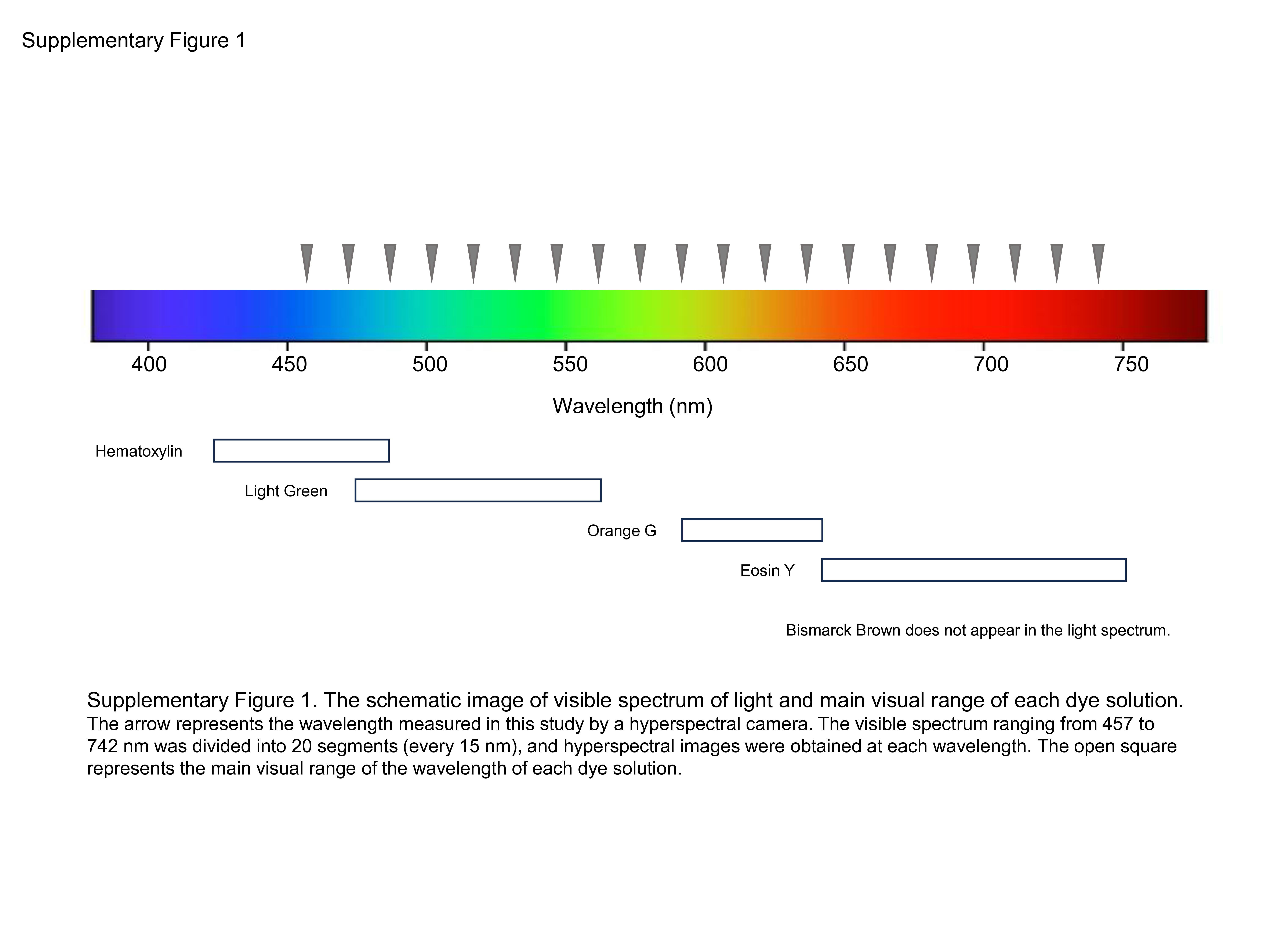

Supplement: Supplementary file 1 — Supplementary Figure S1: Supporting information. [file CAM4-15-e71746-s002.tif]
